# Lactate regulators contribute to tumor microenvironment and predict prognosis in lung adenocarcinoma

**DOI:** 10.3389/fimmu.2022.1024925

**Published:** 2022-11-25

**Authors:** Shipeng Shang, Mi-zhou Wang, Zhiyuan Xing, Ningning He, Shangyong Li

**Affiliations:** ^1^ School of Basic Medicine, Qingdao University, Qingdao, China; ^2^ Anesthesia Operating Department, The Affiliated Qingdao Municipal Hospital of Qingdao University, Qingdao, China; ^3^ Department of Abdominal Tumor Surgery, Qingdao Central Hospital to Qingdao University, Qingdao, China

**Keywords:** lactate regulator, lung adenocarcinoma, cancer prognosis, risk model, immunotherapy

## Abstract

**Background:**

Lactic acid, as a product of glycolysis, increases tumor cell migration and the invasion of tumor cells in the tumor microenvironment. Besides this, lactic acid promotes the expression of programmed death-1 expression (PD-1) in regulatory T cells, which could cause the failure of PD-1 blockade therapy. However, the implications of lactic acid in the tumor microenvironment of lung adenocarcinoma (LUAD) remain largely unclear.

**Methods:**

We performed unsupervised consensus clustering to identify lactic-associated subtypes using expression profile of lactate regulators in LUAD. Differentially expressed genes (DEGs) associated with lactic-associated subtypes was used to construct lactate signature (LaSig) using LASSO regression algorithm. Immune infiltration analysis was conducted by ESTIMATER and drug sensitivity was estimated by R package called “pRRophetic”. The difference between two groups was calculated using Wilcox rank sum test and correlation analysis was calculated using Pearson correlation coefficient.

**Results:**

In this study, we evaluated DNA methylation and the mutation frequency of lactate regulators and found lactate regulators showed low mutation frequency in the TCGA-LUAD cohort, except TP53. At the RNA level, the expression level of lactate regulators was significantly associated with the immune cell component. In particular, expression of LDHA was positively correlated with CD4 T cell, CD8 T cell, M1 macrophages, and the enrichment score of multiple immune pathways. Two clusters were defined using the gene expression level of lactate regulators, and LDHA was significantly upregulated in cluster 1 with poor overall survival. A lactate signature (LaSig) had a robust performance in predicting the survival rate and immunotherapy response of LUAD patients. Moreover, patients in the high LaSig group may be more likely to benefit from these drugs (Cisplatin, Erlotinib, Gemcitabine, and Vinblastine) than those in the low LaSig group.

**Conclusion:**

In summary, our study explores the role of lactate regulators in guiding the clinical treatment of lung adenocarcinoma and provides additional help to supplement traditional molecular subtypes.

## Introduction

The Warburg effect is an important metabolic feature of tumors, and it rapidly generates energy through aerobic glycolysis (1, 2). Unlike normal cells, tumor cells can produce lactic acid with sufficient oxygen to fuel tumor cells, which contributes to the tumor invasion and metastasis (3). In previous studies, lactate production is demonstrated to be closely associated with the growth of a variety of cancers, including lung (4), breast (5), and gastric cancer (6). The lactate dehydrogenase-A (LDHA) enzyme is found to play an essential role in the survival and proliferation of cancer cells (7). Besides this, the antiviral and antitumor functions of natural killer cells were enhanced by LDHA (8).

Lung adenocarcinoma (LUAD) is the most common type of lung cancer and a deadly malignant tumor with high mortality (9). Immunotherapy has become an important therapeutic strategy for LUAD with low response rates because of tumor heterogeneity and adverse events (10, 11). Identifying effectiveness biomarkers is essential to improve the effect of immunotherapy. Currently, a variety of biomarkers are used to evaluate the response of immunotherapy, including tumor mutation burden (12), PD-1, PD-L1, CTLA-4 (13), TIGIT (14), MSI (15), and Neoantigen (16). The complex immune microenvironment is an important factor that leads to the different immunotherapy responses of cancer patients. The significant characteristic of the tumor microenvironment is hypoxia, leading to an elevated level of lactic acid produced by cancer cells. The establishment of an immunosuppressive environment is closely related to metabolites (such as lactic acid), which can promote immune escape in the tumor microenvironment (17). In addition, lactic acid plays a vital role in the tumor microenvironment by regulating T cells and can promote the expression of PD-1, which is of great significance for immunotherapy (18). The increase of lactic acid can promote the activity of myeloid-derived suppressor cells and promote the activity of tumor cells (6). However, the study of lactic acid–related in the tumor environment is still limited. The regulating effect of lactic regulators needs to be analyzed in LUAD.

In this study, we aimed to analyze the relationship between the lactic regulator and the immune environment. The established LaSig scoring tool was used to predict prognosis and immunotherapy response in LUAD. LaSig had robust predictive performance and robustness in prognosis of LUAD and played a role in predicting drug sensitivity. In addition, LaSig can be used as a potential marker to predict prognosis of pan-cancer patients. Our results indicate that the lactic regulator may serve as biomarker of prognosis and immunotherapy response of LUAD.

## Methods

### Data collection and processing

Lactate-associated genes were collected from GO terms in the Molecular Signatures Database (MiSigDB). TCGA gene expression data, DNA methylation data, somatic mutation data, copy number variation (CNV) data, and clinical information were downloaded from Xena public data hubs (https://xenabrowser.net/).

Gene expression data of the additional LUAD samples were obtained from the Gene Expression Omnibus (GEO) database (including GSE31210 and GSE19188). Ensemble ID was converted to a gene symbol, and expression levels of genes containing more than one ensemble ID were represented by the average value. The gene expression level of TCGA-LUAD was expressed in transcripts per million (TPM). The probes were converted to gene symbols based on the annotation file of the Affymetrix Human Genome U133 Plus 2.0 Array. Immunotherapy-associated data of LUAD samples were downloaded from GSE126044 and GSE135222 (**Table 1**).

**Table 1:** Relevant information for all data sets in this study.

Dataset	Platform	Number of Samples (Numbers of Cancer tissue)
TCGA-LUAD	Illumina HiSeq	585 (526)
GSE31210	GPL570	246 (246)
GSE19188	GPL570	156 (36)
GSE126044	GPL16791	16 (16)
GSE135222	GPL16791	27 (16)

### Unsupervised consensus clustering

To identify lactic-associated subtypes, unsupervised clustering was performed to cluster tumor samples into subtypes according to the expression matrix of lactic- associated genes. A consistency clustering algorithm was performed by using the “ConsensusClusterPlus” R package, and it was repeated 1000 times (19).

### Generation of the LaSig score

First, differentially expressed genes (DEGs) between clusters 1 and 2 were identified using the “limma” R package with a threshold of |log2FC|>1 and adjusted *p* value<.01. Second, LUAD samples were randomly divided into training and testing sets according to a ratio of 2:1. Univariate Cox regression analysis of these genes was performed to look for the survival-associated signatures in LUAD, and genes with *p*-value<.05 were selected for further analysis. Then, the LASSO regression model and 10-fold cross-validation were performed to reduce the dimensionality and select representative genes by using the “glmnet” R package. Finally, we selected 25 genes, and their coefficients were used to generated the LaSig score by the following formula:


$$\text{LaSig Score }=\;\sum_{i}^{n}Gene_{i}*Coef_{i}$$

where *Gene_i_* and *Coef_i_* represent the expression level and LASSO coefficient of each selected gene, respectively.

### Gene set enrichment analysis

GO and KEGG enrichment analysis of DEGs were performed using the “clusterProfiler” R package (20). Seventeen immune pathway–associated genes were collected from The Immunology Database and Analysis Portal (ImmPort) database (https://www.immport.org/). The immune pathway score of LUAD samples was calculated by the “GSVA” R package.

### Tumor microenvironment estimation

Subpopulations of 22 immune cells were estimated by using CIBERSORTx (http://cibersort.stanford.edu/) with the gene expression profile of LUAD samples (21). The samples with *p<*.05 were employed for further analysis.

### Analysis of drug sensitivity

An R package called “pRRophetic” was used to estimate drug sensitivity. Fifty percent of cellular growth inhibition (IC_50_) was used as an indicator of drug sensitivity.

### Statistical analysis

The Pearson correlation coefficient was used for correlation analysis. The Wilcox rank sum test was used to calculate the difference between the two groups. The Kaplan–Meier method was used to compare the overall survival of LUAD patients. All statistical analyses were conducted using R (R 4.1.2) software and *p*<.05 was considered significant.

## Result

### Multi-omics feature of lactic in LUAD

**Figure 1:**
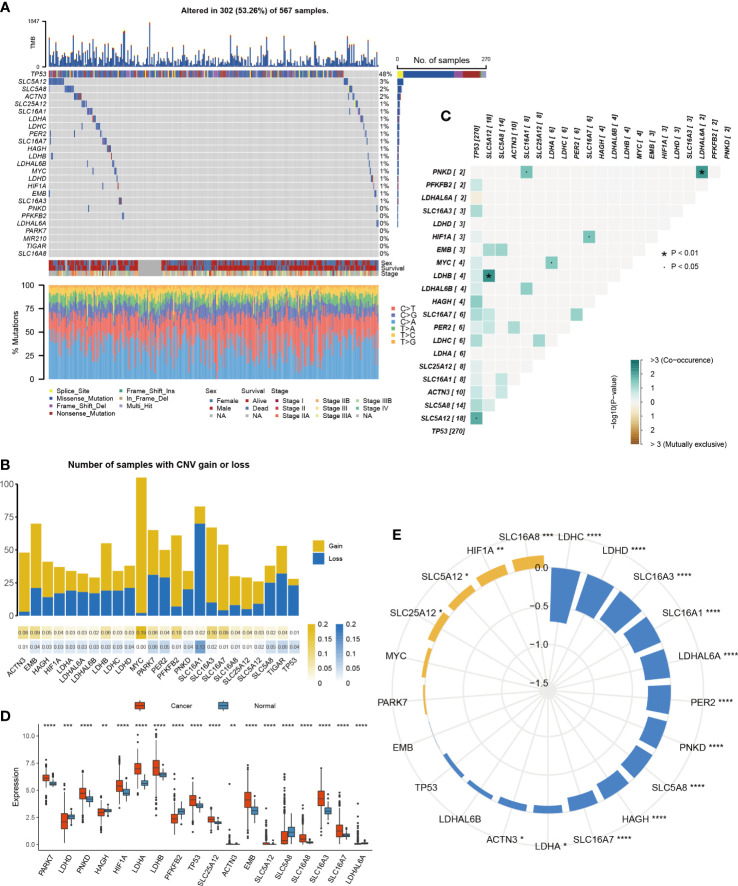
Multi-platform features of lactic regulators in LUAD. **(A)** Mutation of lactic regulators in TCGA-LUAD cohort. **(B)** Co-occurrence feature of lactic regulators in TCGA-LUAD cohort. **(C)** Copy number variation frequency of lactic regulators in TCGA-LUAD cohort. Yellow stripe represents copy number gain, and blue stripe represents copy number deletion. **(D)** Comparison of gene expression of lactic regulators between LUAD and normal tissue. **(E)** Correlation of DNA methylation and gene expression of lactic regulators in TCGA-LUAD cohort. Yellow represents positive correlation, and blue represents negative correlation (*P<.05; **p<.01; ***p<.001; ****p<.0001).

To evaluate the influence of lactic acid on LUAD, 25 lactic regulators were summarized by KEGG pathway. First, the mutation profiles of LUAD patients were studied, and we found that, except the *TP53*, mutations in 21 lactic regulators were rare in LUAD, ranging from 0% to 3% (**Figure 1A**). Next, the co-occurrence feature of lactic regulators was analyzed, *SLC5A12* and *LDHB*, *PNKD* and *LDHAL6A* have a co-occurrence relationship (**Figure 1B**). Besides this, *ACTN3*, *HAGH*, *LDHA*, and *LDHAL6A* were more likely to have copy number gains. Conversely, *TP53*, *LDHAL6B*, and *MIR210* were more likely to have copy number deletions (**Figure 1C**).

The difference in lactic regulators between LUAD and normal lung tissue were studied. Compared with normal tissue samples, 18 of 25 lactic regulators were aberrantly expressed in tumor samples (**Figure 1D**). To analyze the effect of DNA methylation on gene expression of lactic regulators, correlation between DNA methylation and gene expression was calculated. DNA methylation was negatively correlated with the gene expression level of *ACTN3*, *HAGH*, *LDHA*, *LDHAL6A*, *LDHC*, *LDHD*, *PER2*, *PNKD*, *SLC16A1*, *SLC16A3*, *SLC16A7*, and *SLC5A8* (**Figure 1E**).

These results reveal the multi-omics characteristics of the lactate regulatory factor in LUAD. At the RNA and epigenetic levels, most of the lactic regulators showed an abnormal pattern in tumor tissue compared with normal tissue, and DNA methylation may affect the gene expression of lactic regulators.

### Prognosis and immune characteristics of lactic regulators

**Figure 2:**
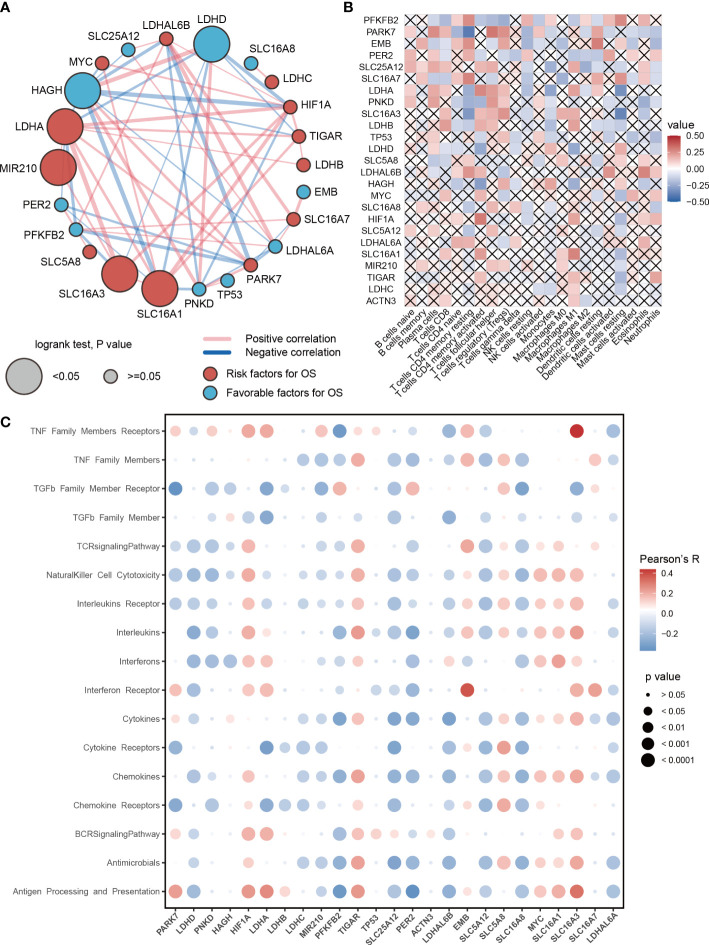
Prognosis and immune characteristics of lactic regulators. **(A)** Correlations and prognosis value of DNA lactic regulators in TCGA-LUAD cohort. **(B)** Correlation heat map between lactic regulators and 22 immune cells. Red indicates positive correlation; blue indicates negative correlation; cross indicates p>=.05. **(C)** The correlation between expression level of lactic regulators and immune-associated pathway.

To further study the role of lactic regulators in LUAD, a univariate Cox regression model was used to estimate the prognosis value of these lactic regulators. High expression of *LDHA*, *SLC16A1*, *SLC16A3*, and *MIR210* were risk factors of overall survival for LUAD; on the contrary, high expression of *HAGH* and *LDHD* were protective factors (**Figure 2A**). In addition, *HAGH* and *LDHD* had a relatively strong positive correlation in RNA level.

Recent studies show that lactic acid plays an important regulatory role for immune cells in tumors (22, 23). Therefore, we investigated the relationship between lactic regulators and the immune cell. The expression level of *PFKFB2* and *PARK7* were associated with the abundance of 2/3 immune cells (**Figure 2B**). Moreover, lactic regulators were significantly correlated with multiple immune pathways (**Figure 2C**). The expression level of *EMB* and *SLC16A3* were positively correlated with interferon receptors and members of the TNF family of receptors, respectively. In summary, the expression of *PARK7*, *LDHD*, *PNKD*, *HAGH*, *MIR210*, *PFKFB2*, *PER2*, *SLC5A12*, and *SLC16A8* had a negative correlation with the pathway activity of the T cell receptor signaling pathway. The expression of HIF1A, TIGAR, EMB, SLC5A8, MYC, SLC16A1, and SLC16A7 was positively correlated with the enrichment score of the T-cell receptor signaling pathway

### Construction of lactate-associated signatures

**Figure 3:**
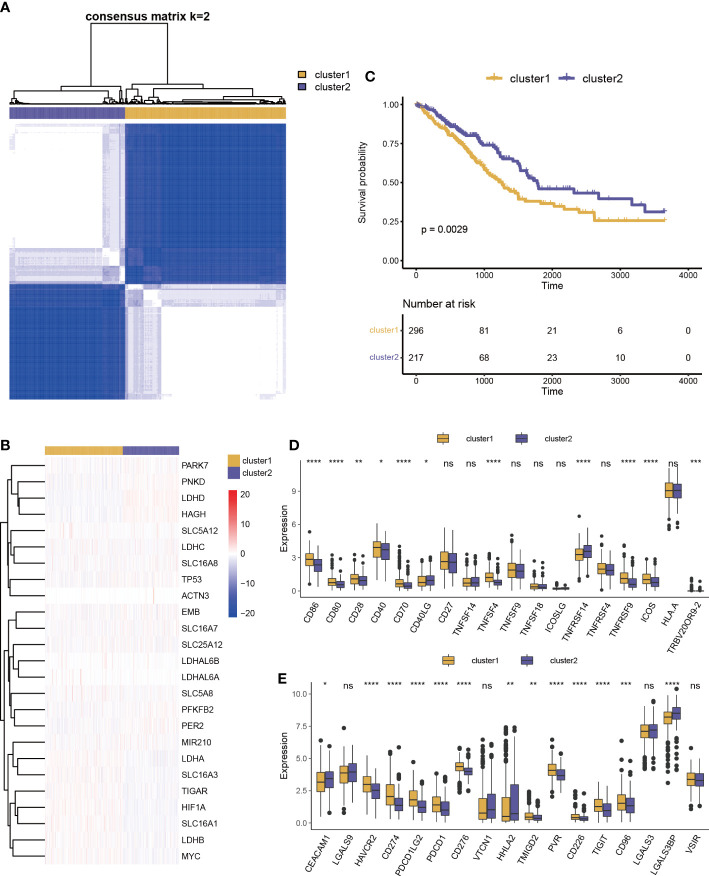
Prognosis and immune characteristics of lactic regulators. **(A)** Consensus clustering analysis of lactic regulators for optimal *k* = 2. **(B)** Kaplan–Meier curves of OS for two clusters of patients. **(C)** Heat map of 25 lactic regulators between the two distinct subtypes. **(D, E)** Differential analysis of costimulatory and coinhibitory molecules two clusters (*p<.05; **p<.01; ***p<.001; ****p<.0001).

Lactic regulators may have important contributions to tumor heterogeneity due to their close links with the immune cell and immune pathway. LUAD samples were clustered into two categories using unsupervised clustering (**Figure 3A**). There were 13 genes with high expression levels in cluster 1 and 12 genes with high expression levels in cluster 2 (**Figure 3B**). As shown in **Figure 3C**, there is a significant difference in survival rate between the two groups. This result suggests that lactic regulators may further influence patient survival by mediating immune pathways.

We collected costimulatory and coinhibitory molecules from the work of Kim et al *(* 24) and compared differences of their expression levels between the two clusters. Multiple costimulatory molecules, such as *CD86*, *CD80*, *CD28*, *CD40*, *CD70*, *TNFSF4*, *TNFRSF9*, *ICOS*, and *TRBV20OR9-2*, showed a higher expression level in cluster 1 (**Figure 3D**). Multiple coinhibitory molecules, such as *HAVCR2*, *CD274*, *PDCD1LG2*, *PDCD1*, *VSIR*, *CD276*, *TMIGD2*, *PVR*, *CD226*, *TIGIT*, and *CD96*, also showed a higher expression level in cluster 1 (**Figure 3E**).

**Figure 4:**
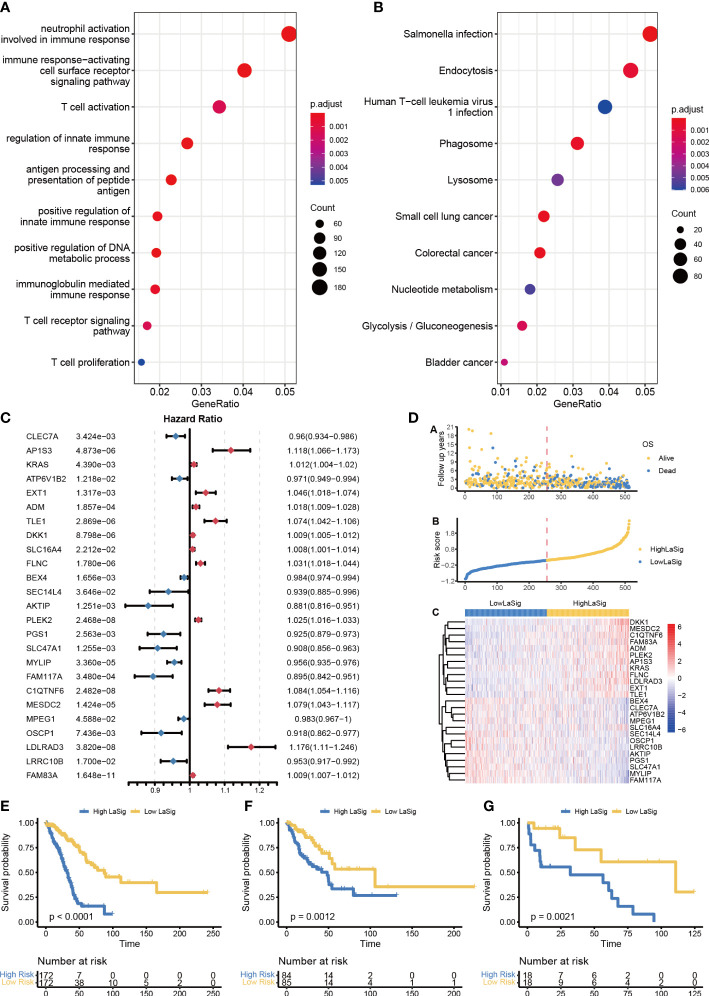
Construction of LaSig to predict the prognosis of LUAD patients. **(A, B)** GO and KEGG pathway enrichment analysis of differential expression genes. **(C)** The forest plot of key lactic signatures using univariate Cox regression analysis. **(D)** The distributions of risk scores, OS status, and gene expression of key lactic signatures. **(E–G)** Kaplan–Meier curves of high and low risk groups in training set €, test set **(F)**, and GSE19188-cohort **(G)**.

The impact of lactic regulators for tumor heterogeneity was further explored, and we identified 4318 DEGs between clusters 1 and 2. These genes were enriched in immune-related terms by using GO analysis and cancer-related terms by using KEGG pathway analysis (including immune response−activating cell surface receptor signaling pathway, neutrophil activation involved in immune response, and Salmonella infection; **Figures 4A, B**). Univariate Cox regression analysis was used to select a prognosis-associated gene, and expression levels of 1007 genes were found to be significantly associated with survival. Twenty-five key genes were selected to construct LaSig by using a LASSO regression model and tenfold cross-validation in the training set (**Figure S1**). The formula of LaSig was: (-0.179)*CLEC7A+(0.008)*AP1S3+(0.044)*KRAS+(-0.067)*ATP6V1B2+(0.023)*EXT1+(0.014)*ADM+(0.078)*TLE1+(0.057)*DKK1+(0.011)*SLC16A4+(8.37e-6)*FLNC+(-0.04)*BEX4+(-0.008)*SEC14L4+(-0.023)*AKTIP+(0.084)*PLEK2+(-0.073)*PGS1+(-0.014)*SLC47A1+(-0.112)*MYLIP+(-0.067)*FAM117A+(0.139)*C1QTNF6+(0.143)*MESDC2+(-0.005)*MPEG1+(-0.042)*OSCP1+(0.296)*LDLRAD3+(-0.075)*LRRC10B+(0.011)*FAM83A. In the low LaSig group, the high expression of 12 genes is a risk factor for LUAD, and the high expression of 13 genes is a protective factor. The high LaSig and low risk groups were divided according to the median value of LaSig (cutoff of training and testing sets: 0.117 and 0.007). There was a significant difference in survival between high and low risk groups in the training set, validation set, and GSE19188 (**Figures 4E–G**). This suggests the role of LaSig in predicting survival of LUAD patients.

To assess the relationship between LaSig and clinical features, we compared the age, gender, and stage of LUAD patients in the high LaSig and low risk groups. We found that T4, N2, M1, and stage have higher LaSig scores (**Figure S2**). This suggests LaSig may reflect the malignancy degree of the tumor.

### Drug sensitivity between high and low LaSig group patients

**Figure 5:**
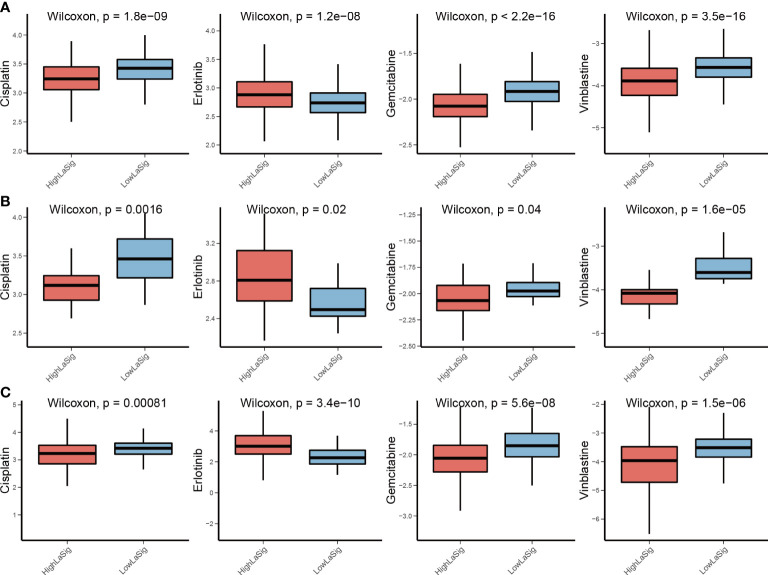
Drug sensitivity comparison between LaSig groups. **(A–C)** Difference comparison of IC_50_ of Cisplatin, Erlotinib, Gemcitabine, and Vinblastine between high and low LaSig groups in the TCGA **(A)**, GSE19188 **(B)**, and GSE31210 cohorts **(C)**.

Chemotherapy is widely used in the treatment of LUAD. However, cancer patients have different drug sensitivity due to tumor heterogeneity. We compared IC_50_ of high and low LaSig group patients to find out whether LaSig score is applicable to personalized treatment strategies. The patients in the high LaSig group were sensitive to Cisplatin, Gemcitabine, and Vinblastine, and the patients in the low LaSig group was more sensitive to Erlotinib in the TCGA, GSE31210, and GSE19188 cohorts (**Figure 5**). This may provide help in determining therapeutic strategies for LUAD patients.

### The role of LaSig in predicting immunotherapy response of LUAD

**Figure 6:**
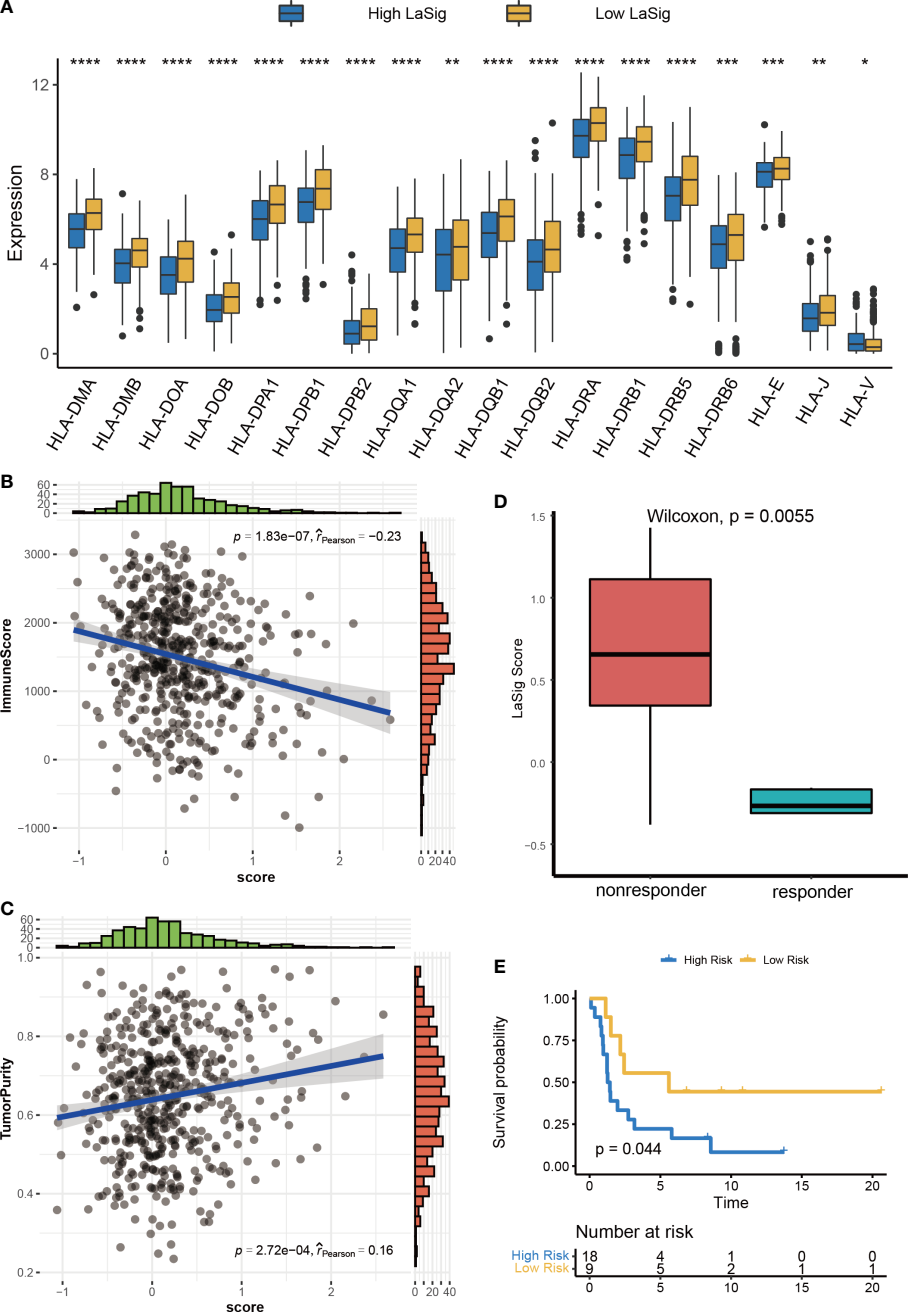
Correlation analysis between LaSig and anti-PD-L1 immunotherapy. **(A)** The expression level of human leukocyte antigen in high and low LaSig groups. **(B)** Correlation between LaSig and tumor purity. **(C)** Correlation between LaSig and stromal score. **(D)** LaSig score of patients with different immunotherapy responses. **(E)** Survival analysis of patients with different LaSig groups (*p<.05; **p<.01; ***p<.001; ****p<.0001).

The above results reveal the close relationship between lactic regulators and the immune microenvironment. We further explored the role of LaSig score in guiding immunotherapy response. First, the human leukocyte antigen had higher expression level in low LaSig than high LaSig (**Figure 6A**). Second, tumor purity and the immune score of LUAD patients were calculated. LaSig was negatively correlated with tumor purity and positively correlated with immune score in LUAD (**Figures 6B, C**). Second, to evaluate the role of LaSig score in predicting immunotherapy response, the LaSig score of non–small cell lung cancer patients treated with anti-PD-1/PD-L1 was calculated. We found that LaSig scores of nonresponders were significantly higher than those of responders (**Figure 6D**). Besides this, the patients were divided into two groups by using the LaSig score cutoff, and the low LaSig score group had a better prognosis (**Figure 6E**). These results reveal the potential role of LaSig in predicting immunotherapy.

### Exploring the role of LaSig in the pan-cancer cohort

**Figure 7:**
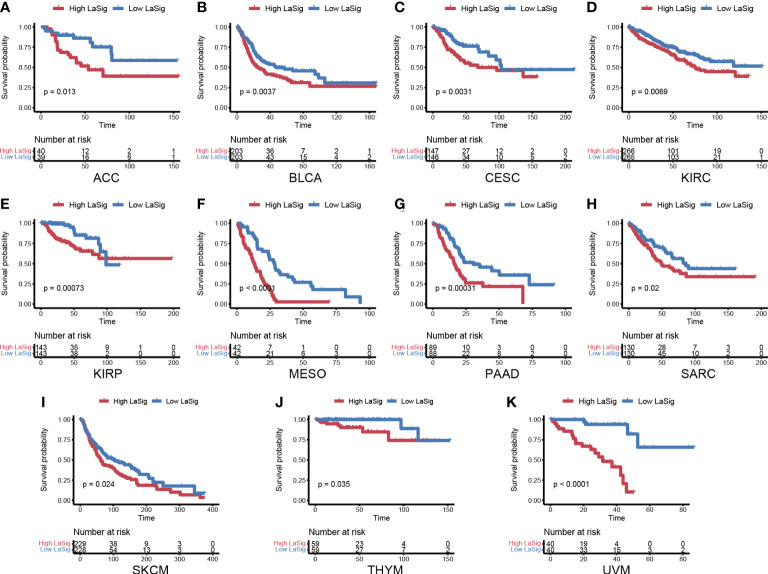
The role of LaSig in the pan-cancer cohort. **(A–K)** Survival analysis of LaSig in pan-cancer dataset.

We next studied the role of LaSig in predicting the prognosis of the pan-cancer cohort. LaSig was significantly associated with prognosis in 11 cancer types (**Figure 7**), including adrenocortical cancer (ACC), bladder cancer (BLCA), cervical cancer (CESC), kidney clear cell carcinoma (KIRC), kidney papillary cell carcinoma (KIRP), mesothelioma (MESO), pancreatic cancer (PAAD), sarcoma (SARC), melanoma (SKCM), thymoma (THYM), and ocular melanomas (UVM). Moreover, LaSig also represented the expression of PD-1, which is significantly positively correlated with the expression of PD-1, including BLCA, kidney chromophobe (KICH), acute myeloid leukemia (LAML), lower grade glioma (LGG), liver cancer (LIHC), LUAD, pancreatic cancer (PAAD), testicular cancer (TGCT) and UVM (**Figure S3**).

## Discussion

Lactic acid has long been considered as metabolic waste of highly proliferating cells. Nevertheless, lactic acid recently has been found to be an important product affecting tumor proliferation and metastasis (25, 26). Lactic acid could regulate T cell migration and effector function and promote the expression of PD-1 (27). However, the impact of lactic acid in the immune microenvironment of LUAD has not been identified clearly. To explore the role of lactate regulators in the immune microenvironment of LUAD can help us understand the effect of lactic acid on LUAD and guide immunotherapy.

In this study, 25 lactate regulators were collected and analyzed in LUAD. The expression level of a large number of lactate regulators in LUAD samples changed. DNA methylation of lactate regulators has a significant negative correlation with the expression of genes, which demonstrates that DNA methylation regulates expression of those genes that were associated with abnormal metabolism of the tumor. The acidification of the tumor microenvironment is an important cause of carcinogenesis processes, including metastasis and immune escape (28). The increase in lactate in the tumor is more consistent with tumor growth and migration.

Lactate regulators are also significantly correlated with immune cells, and PARK7 was negatively correlated with resting memory CD4+ T cell. In addition, the increased levels of extracellular lactate are closely associated with the Notch1/TAZ axis, which can inhibit the activity of cytotoxic T cells and lead to the proliferation and migration of lung cancer cells (29). Thus, PARK7 as a redox-sensitive chaperone may affect the status of the CD4+ T cell.

Two groups were obtained by unsupervised cluster analysis of gene expression levels of lactic acid regulators, which can distinguish prognosis. DEGs were identified between two clusters and mainly enriched in immune- and cancer-related pathways. These results suggest that molecular subtypes based on the expression level of lactate regulators may be an important prognostic feature in cancer patients. We constructed and validated a prognosis risk signature with 25 lactate regulator–related genes, named LaSig, which divided LUAD patients into high and low LaSig groups. The level of HLA gene expression and immune score in the low LaSig group were higher than those in the high LaSig control group. Hence, the immunotherapy data set is further used to verify the predictive value of LUAD immunotherapy response. Heterogeneity of the tumor microenvironment is an important factor affecting the treatment of cancer patients, including chemotherapy and immunotherapy. The difference of lactate metabolism is one of the reasons for the heterogeneity of the tumor microenvironment. Alteration of the tumor metabolism may be a potential solution to improve the efficacy of immunotherapy. In addition, LaSig also has predictive ability of prognosis in many types of cancer.

## Conclusion

In this study, we analyzed the association between lactate regulators and immune cells. The LaSig score was constructed to predict prognosis and immunotherapy response of LUAD. LaSig may become a valuable signature to guide the treatment of LUAD patients. The expression level of lactate regulators is associated with immune cells and the immune checkpoint in the tumor environment. The prognostic risk model based on multiple lactate signature genes provides a new perspective for predicting prognosis and immunotherapy response.

## Data availability statement

The datasets presented in this study can be found in online repositories. These datasets were downloaded from TCGA database, GSE31210, GSE19188, GSE126044 and GSE135222.

## Author contributions

NH and SL designed and directed all the research. SS performed the experimental analysis and drafted the manuscript. M-ZW revised the manuscript. ZX participated in language editing. All authors contributed to the article and approved the submitted version.

## Funding

This study was supported by the Shandong Provincial Youth Entrepreneurship Program for Colleges and Universities (2021KJ075).

## Acknowledgments

We thank the members of the research team for their support and guidance.

## Conflict of interest

The authors declare that the research was conducted in the absence of any commercial or financial relationships that could be construed as a potential conflict of interest.

## Publisher’s note

All claims expressed in this article are solely those of the authors and do not necessarily represent those of their affiliated organizations, or those of the publisher, the editors and the reviewers. Any product that may be evaluated in this article, or claim that may be made by its manufacturer, is not guaranteed or endorsed by the publisher.
