# Supplementary material for: Lactate regulators contribute to tumor microenvironment and predict prognosis in lung adenocarcinoma
Source: Front Immunol. 2022 Nov 25;13:1024925. doi: 10.3389/fimmu.2022.1024925 (PMC9732022; doi:10.3389/fimmu.2022.1024925)
Supplement: Supplementary file 1 [file DataSheet_1.docx]

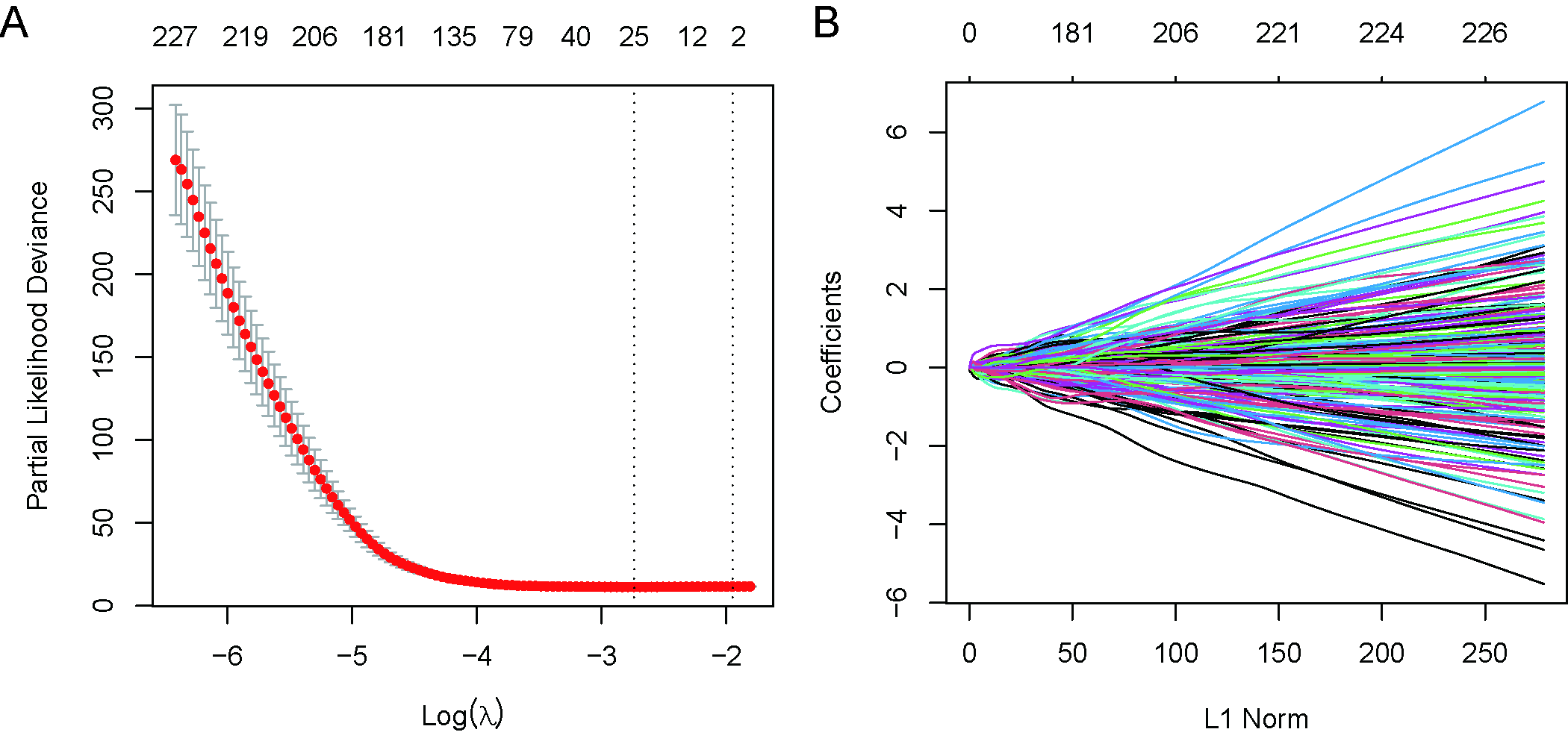


**Figure S1. Feature selection using** **LASSO model.** (A) LASSO analysis of lactate signature with minimum lambda value. (B) Coefficient of lactate signature in LASSO model.


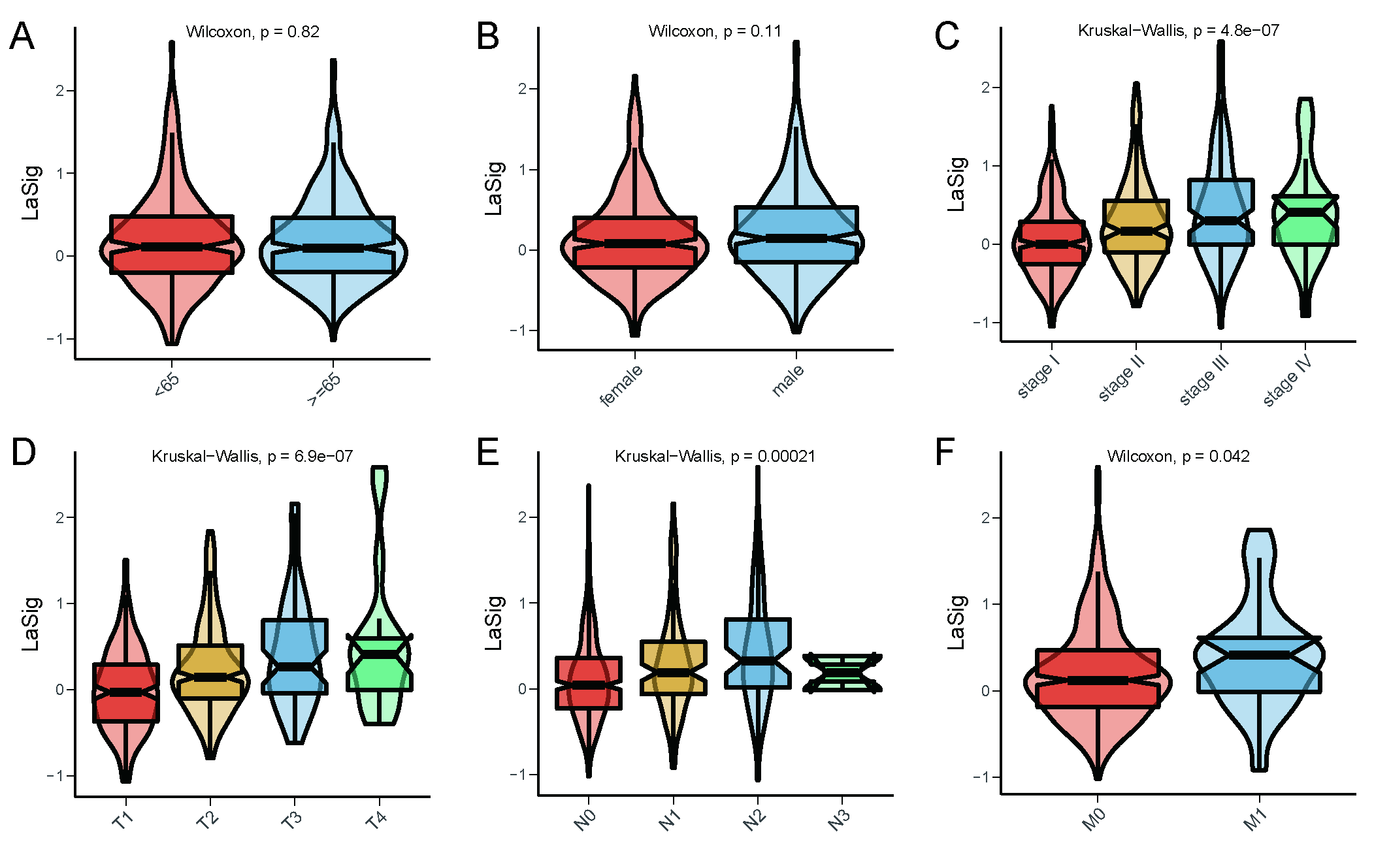


**Figure S2. Comparison of LaSig among different clinical features.**


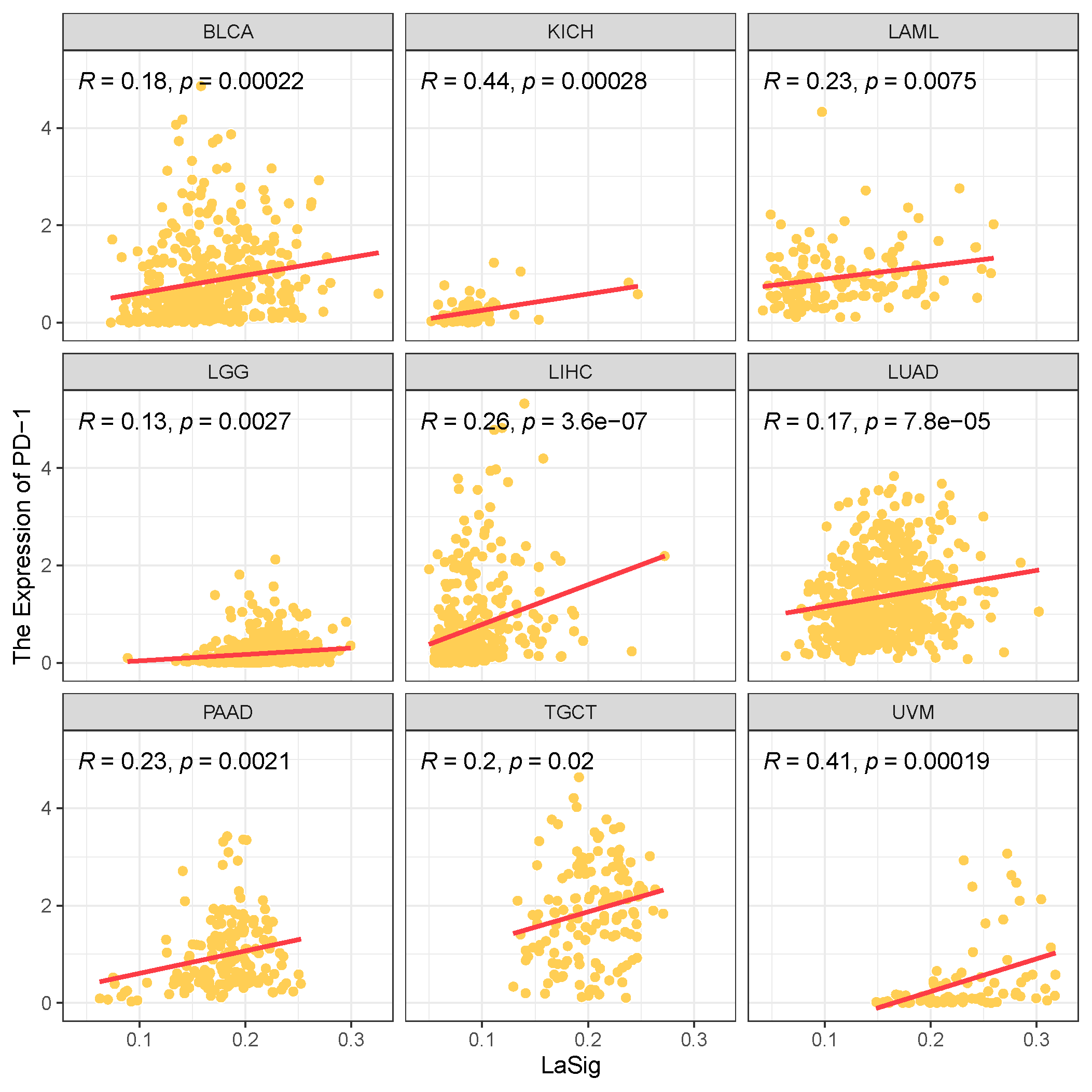


**Figure S3. Association analysis of LaSig and PD-1 expression in different cancers.**


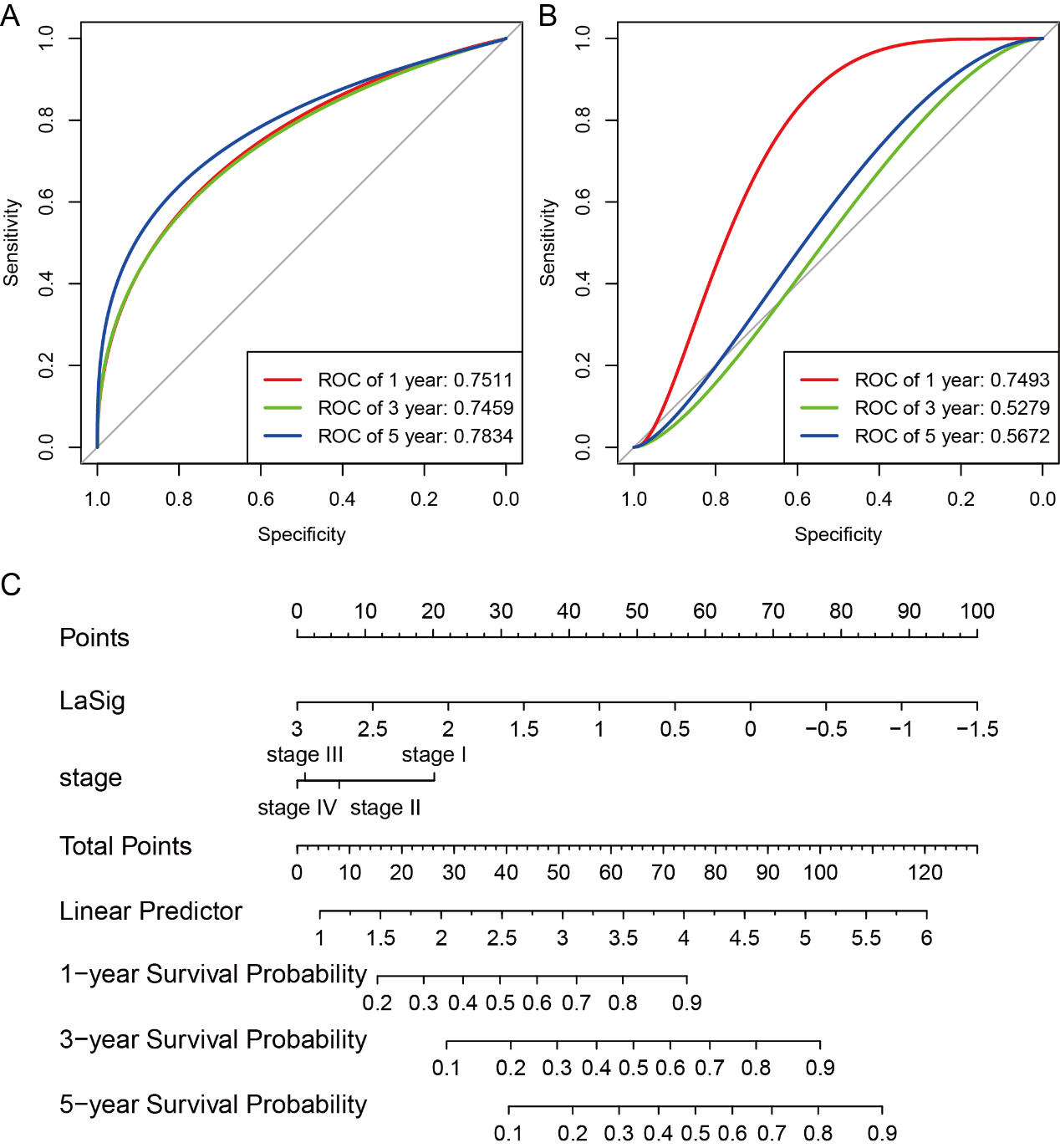


**Figure S3. LaSig incorporates clinicopathological features to further improve prognostic models.** (A-B) ROC curve was used to evaluate the predictive efficacy of LaSig in TCGA-LUAD and GSE19188. (C) Nomograms for forecasting the 1 -, 3 -, and 5-year patient survival rate using the TCGA-LUAD dataset.
